# Standardized Loads Acting in Knee Implants

**DOI:** 10.1371/journal.pone.0086035

**Published:** 2014-01-23

**Authors:** Georg Bergmann, Alwina Bender, Friedmar Graichen, Jörn Dymke, Antonius Rohlmann, Adam Trepczynski, Markus O. Heller, Ines Kutzner

**Affiliations:** 1 Julius Wolff Institute, Charité – Universitätsmedizin Berlin, Berlin, Germany; 2 Engineering Science Unit, University of Southampton, Highfield, Southampton, Great Britain; Van Andel Institute, United States of America

## Abstract

The loads acting in knee joints must be known for improving joint replacement, surgical procedures, physiotherapy, biomechanical computer simulations, and to advise patients with osteoarthritis or fractures about what activities to avoid. Such data would also allow verification of test standards for knee implants. This work analyzes data from 8 subjects with instrumented knee implants, which allowed measuring the contact forces and moments acting in the joint. The implants were powered inductively and the loads transmitted at radio frequency. The time courses of forces and moments during walking, stair climbing, and 6 more activities were averaged for subjects with I) average body weight and average load levels and II) high body weight and high load levels. During all investigated activities except jogging, the high force levels reached 3,372–4,218N. During slow jogging, they were up to 5,165N. The peak torque around the implant stem during walking was 10.5 Nm, which was higher than during all other activities including jogging. The transverse forces and the moments varied greatly between the subjects, especially during non-cyclic activities. The high load levels measured were mostly above those defined in the wear test ISO 14243. The loads defined in the ISO test standard should be adapted to the levels reported here. The new data will allow realistic investigations and improvements of joint replacement, surgical procedures for tendon repair, treatment of fractures, and others. Computer models of the load conditions in the lower extremities will become more realistic if the new data is used as a gold standard. However, due to the extreme individual variations of some load components, even the reported average load profiles can most likely not explain every failure of an implant or a surgical procedure.

## Introduction

### Why are standard loads needed?

Knowledge of contact forces and moments acting in the tibio-femoral joint is needed for testing wear, fatigue, or strength of implants, for analyses of strain distribution and remodeling at the fixation area, and for other purposes. Reliable data can also serve as a ‘gold standard’ for the verification of analytical musculo-skeletal models. Realistic finite element models of natural knee joints including the surrounding soft tissues permit the calculation of the mechanical situation in structures such as cartilage, ligaments, or menisci, for example in cases of injuries, or permit the investigation of the biomechanical consequences of surgical interventions.

Loading of the knee joint primarily depends on the physical activity. It is also determined by body weight (BW), but individually differs greatly, even between subjects with the same BW [1]. This raises the question of which loads are appropriate to use for mechanical tests or analyses. For wear and fatigue those activities are most decisive which cause very high loads and additionally act most frequently. For static strength and fixation stability, even rarely acting extreme loads may additionally be important.

One could determine the load-time patterns during the most strenuous and frequent activities of daily living (ADL) as they act on average in subjects with an average body weight. These activities are walking and climbing stairs [2]. However, the median loads will then be higher in 50% of subjects and 50% of loading cycles, and this would not be adequate for use in strength or wear tests. A more justified approach would be to take data from subjects with a high BW and joint loads which are, relative to the BW, higher than in most other subjects. However, this may cause other problems because such high loads could lead to failures of small implants.

### Calculation of knee contact loads

Contact loads in the knee joint can either be calculated or measured. To calculate the joint forces, kinematic data as well as ground reaction forces serve as input for inverse dynamic musculo-skeletal models. However, substantial variations in the calculated forces exist. In most studies, contact forces of 200–400%BW (percent of the body weight) were calculated for level walking [3]–[8], but forces of 450%BW [9] and even up to 670%BW [10] have also been reported. Potential sources of error for such models are non-validated optimization criteria, insufficient modeling of muscles, and antagonistic muscle activities, amongst others.

### Measurement of knee contact loads

Instrumented implants allow access to the joint contact forces *in vivo*. In previous studies, forces were measured in a distal femur replacement and transformed to the knee joint [11]–[13]. Peak axial forces of 220–250%BW were reported for level walking and 280%BW for descending stairs.

To measure the tibio-femoral contact force directly, instrumented knee implants were also developed by others. An initial design measured the axial force and the center of pressure [14], and a second design enabled the measurement of all six force and moment components [15]. Load data was reported for 1–3 subjects. During walking, forces between 180 and 280%BW were measured [16]. With respect to daily activities, the highest forces, approximately 350%BW, occurred during stair ascending and descending [17]. During all investigated activities, the shear forces were substantially lower than the axial forces [18]. Peak anterior shear forces of 30%BW were observed during walking.

The instrumented knee implant, developed by us, measures the tibio-femoral contact forces and moments *in vivo* [19]. The electronics in the tibial component are powered inductively and transmit the six load components telemetrically at radio frequency with a measuring error below 2%. During the measurements, the patient's activities are video-taped and recorded together with the loads. Additionally, gait data can also be captured. Synchronous load and video data from many activities can be accessed from the free public database www.OrthoLoad.com, including selected data from this study.

The instrumented implant is based on the INNEX knee (Zimmer GmbH, Winterthur, Switzerland), has an ultracongruent tibial insert, and requires sacrificing the cruciate ligaments. It therefore also transfers load components which are taken up by the ligaments in cruciate ligament retaining implants or in the natural knee. If such implants or the native joint are to be tested or analyzed, they have to be modeled by finite elements and compared to models of the instrumented implant, applying the same loads. This would allow separating the fractions of loads transferred by the soft tissues and by the tibial-femoral contact areas.

### Wear test standard ISO 14243

The test standard ISO 14243-1 [20] defines loads for testing wear in knee implants. The axial force, a/p force, and rotation torque can be compared to the load components F*_z_*, F*_y_*, and M*_z_* now measured *in vivo*. ISO only describes the loads during walking. They were obtained 43 to 25 years ago from analytical musculo-skeletal models and gait data [3], [9] and were edited for the test purpose in 2000 [21]. Because the mathematical modeling has much advanced since then, it can be expected that the new *in vivo* data deviate from the ISO loads. This expectation is supported by a comparison of the axial ISO force with the resultant forces during walking, obtained analytically as well as measured in our patients [22]. During the first 60% of the stance phase both loads differed markedly.

### Goals of this study

The goal of this study was to standardize forces and moments acting in knee implants, based on *in vivo* data. These loads should be suitable as a realistic basis for experimental or analytical studies on wear, fatigue, strength, fixation stability, bone remodeling, or soft tissue loading around the implant. Different classes of loads should be defined as: average loads, high loads, and extreme loads of single force or moment components. Furthermore, the loads defined in the wear test standard ISO 14243 should be compared to the measured values. Based on previous measurements, we hypothesized that the ISO loads are much lower than the measured loads.

## Methods

### Ethics Statement

The study was approved by the Charité Ethics committee (EA4/069/06) and registered at the ‘German Clinical Trials Register’ (DRKS00000606). All patients gave written informed consent prior to participating in this study.

### Coordinate system and measured loads

The coordinate system used is fixed relative to a right-sided implant. Its origin is located in the middle of the tibial plateau at the height of the lowest part of the polyethylene insert [1]. The positive force components F_x_ and F_y_ act in lateral and anterior directions, respectively. The axial force component is reported here as -F_z_ (with a negative sign) and always acts distally in the direction of the *implant shaft*. Positive moments M_x_, M_y_, and M_z_ turn clockwise around their axes during flexion, abduction, and outer rotation of the tibia, respectively. Positive values of M_x_/M_y_ can be caused not only by frictional torque but also by a posterior/lateral shift of the axial force -F_z_. The resultant force F_res_ and the resultant moment M_res_ are calculated from their respective components.

If load components have to be transformed from the implant-based system, used here, to a tibia-based system, the slope of the implants must be respected (Table 1). Relative to the long axis of the tibia, the implants are rotated backwards (positively) around the x-axis by the listed slope angles.

**Table 1:** Investigated subjects and postoperative measuring time.

Subject	K1L	K2L	K3R	K5R	K6L	K7L	K8L	K9L	Ø
**Sex**	M	m	m	m	f	f	m	m	—
**Age [years]**	64	74	71	62	67	76	72	76	70
**Body mass [kg]**	105	92	98	96	83	69	79	109	91
**Height [cm]**	177	171	175	175	174	166	174	166	172
**Tibio-femoral**	3.0	5.0	3.5	1.0	4.0	6.5	4.0	7.0	2.4
**angle [degree]**	varus	varus	varus	varus	**valgus**	varus	varus	varus	varus
**Posterior slope [degree]**	5	11	10	7	7	7	11	6	7
**Date 1 [months]**	20	46	8	30	30	21	25	15	24
**Date 2 [months]**	27	23	16	11	12	12	13	12	16

In the following sections, the terms “peak” force, “peak” component, etc. denote absolute or relative minima or maxima and can be positive or negative. The term “load” either indicates a force, a moment, or a combination of force and moment.

### Measurements

8 subjects with instrumented knee implants participated in this study (Table 1). All subjects obtained the implant due to gonarthrosis and had regained good walking abilities at the time the measurements were taken.

Measurements during 7 ADL were performed at 2 postoperative dates (Table 2). The step height of the staircase was 20 cm and the seat height was 45 cm (50 cm for subject K6 L). The subjects walked at a self-selected speed of approximately 4 km/h. Data from jogging at 6 km/h on a treadmill were also collected in the 3 subjects willing to perform this exercise. The jogging data does not allow statistic evaluations, but can serve as a basis for judgment of the severity of the loads during the ADL. Kinematic data was synchronously recorded by 12 cameras (Vicon, Oxford, UK) on the first postoperative date only (Table 1). More trials from the second postoperative date were added to broaden the data basis when searching for the trials with the absolute highest extreme values of F_res_ (PEAK100, see below) or of single components (EXTREME100).

**Table 2:** Investigated activities, numbers of evaluated cycles per subject, average cycle times, and conversion factors C_aver_ and C_peak_.

	Date 1	Date 2	Cycle Time	C_aver_	C_peak_
Activity	Cycles	Cycles	Tc [s]	[1]	[1]
**Walking**	12–21	18–64	1.07	0.58	1.06
**Ascending stairs**	4–7	8–17	1.78	0.53	1.08
**Descending stairs**	4–9	8–17	1.67	0.60	1.05
**Knee bend**	3–7	4–7	7.61	0.57	1.04
**Standing up**	4–6	4–9	2.68	0.54	1.02
**Sitting down**	4–6	4–8	3.56	0.54	1.09
**One-legged stance**	3–6	4–9	8.45	0.58	1.04
**Jogging, 6 km/h on a treadmill**	-	13–20	0.68	0.57	1.07

*Multiplication of the HIGH100 loads with the conversion factor C_aver_ delivers the AVER75 loads. Multiplication of the HIGH100 loads with C_peak_ delivers the PEAK100 loads. T_c_ from date 2.*

For evaluation of the loads during walking, single steps were separated, which started and ended with foot contact. Stair climbing cycles were separated at the force minima during the swing phase. Cycles from all other activities were separated with additional time intervals at the beginning and end of the exercise. Evaluation of data is described in the following sections as performed on the forces. Analogue procedures were applied when analyzing the moments.

### Average and high body weight

An average and a high BW were defined, based on data from large studies conducted on the American [23] and German [24] populations. The given BWs of subjects between 60 and 69 years of age were averaged between the females and males of both studies. The average BW was 74.7 kg and 2.3% of the population had a BW above 101.5 kg. For our study, we defined an average BW of 75 kg and a high BW of 100 kg.

The 3 force and 3 moment components were measured in %BW and %BWm (percent of body weight times meter), respectively. These loads were multiplied by 7.36 (9.81 * 75/100) to convert them to N and Nm, respectively, for subjects with an average BW and by 9.81 for those with a high BW. If average/high loads in subjects with a BW of X kg instead of 75/100 kg need to be known, the data given in N or Nm must be multiplied by X/75 or X/100, respectively.

### Basic averaging method

The basic averaging procedure combined n loading cycles (Table 2). Averaging started on the resultant force F_res_ using the following ‘time warping’ procedure [25] (the software can be downloaded from www.OrthoLoad.com). First, all n cycle durations were standardized to ‘100% cycle’ and an average cycle time T_c_ was determined. Then, the time scales of all of the cycles were deformed non-uniformly in such a way that the squared differences between all of the n time-deformed functions of F_res_, summed over the whole cycle time, became a minimum. The obtained deformation of the time scale of each single cycle is called its ‘warping path’. The arithmetic mean pattern of F_res_ was finally calculated from the deformed patterns of all of the cycles and named the ‘average’ pattern. This method minimizes the sum of the squared differences of F_res_ between the cycles evenly over the whole cycle time and preserves the typical characteristics of the analyzed patterns as their extreme values. If, for example, a relative force maximum occurs in only 50% of the n cycles, but at strongly varying times, half of its average height will be present at an average time in the final curve.

Determination of the warping paths by analysis of F_res_ was chosen because the characteristics of all 3 force components, as relative extrema, and their locations within the loading cycles are inherent in the force-time pattern of F_res_.

The warping path of each cycle, obtained by the described analysis of F_res_, was then applied to the belonging 6 load components so that they maintained their synchronization. From the time-deformed components of the n cycles, their arithmetic mean patterns were calculated. This averaging process was performed on load data which had been normalized to each subject's individual body weight.

### Average loads ‘AVER75’ for subjects with average body weight

**Figure 1:**
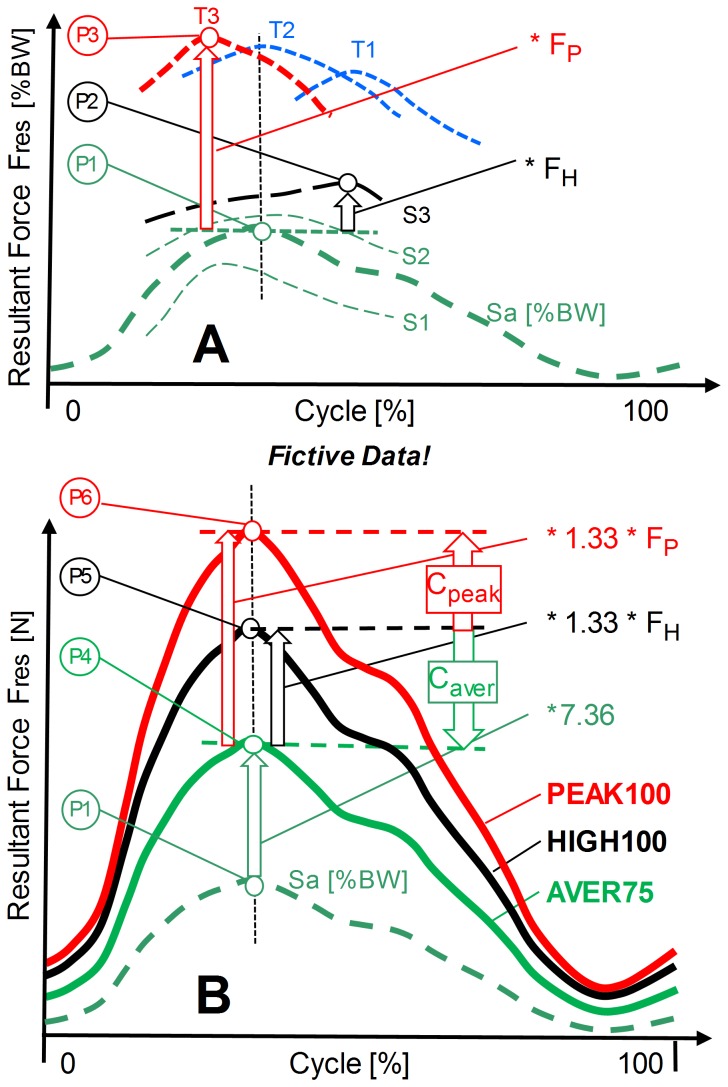
Determination of average, high, and peak forces. Schematic illustration with fictive data from 3 subjects. **Top (A):** S1 to S3 = intra-individual averages in %BW. Curve with P1 = inter-individual average of S1 to S3. Curve with P2 = highest intra-individual average of any of the subjects. F_H_ = multiplication factor between P2 and P1 for calculation of HIGH100 from AVER75 values. T1 to T3 = 3 single trials with highest peak values. Curve with P3 = trial with the highest peak value ever measured. F_P_ = multiplication factor between P3 and P1 for calculation of PEAK100 from AVER75 values. **Bottom (B):** curve Sa (in %BW!) from the top diagram. Curve with P4 = **AVER75** = average load in N for the BW = 75 kg. Curve with P5 = **HIGH100** = high force in N for the BW = 100 kg. Curve with P6 = **PEAK100** = peak force in N for the BW = 100 kg. F_H_ and F_P_ = factors for calculation of HIGH100 and PEAK100 values from AVER75 values. C_aver_ and C_peak_ = multiplication factors for calculation of AVER75 and PEAK100 values from HIGH100 values.

The resultant forces F_res_ from several loading cycles of each subject were first averaged intra-individually (curves S1 to S3 in Figure 1A). The cycles obtained from the 8 subjects were then averaged inter-individually in %BW (curve Sa with the peak value P1 in Figure 1A) and the obtained loads were finally re-calculated for a BW of 75 kg by multiplication with 7.36 (9.81*75/100; curve with the peak value P4 in Figure 1B). This procedure delivered the force pattern AVER75, which represents the average force in subjects with a BW of 75 kg. Identical procedures were applied to all force and moment components.

### High loads HIGH100 for subjects with high body weight

The AVER75 pattern (curve with the peak value P4 in Figure 1B) was multiplied by 1.33 * F_H_. The factor 1.33 increased the BW to the high value of 100 kg. The additional factor F_H_ was the quotient between the highest intra-individual average found in any of the subjects (P2 in Figure 1A) and the inter-individual average of all of the subjects (P1 in Figure 1A). The obtained HIGH100 loads can act in subjects with a BW of 100 kg (e.g. in 1 out of 8 subjects in our study). All factors were applied in the same way on all load components in the AVER75 data.

The HIGH100 loads acted *on average* in 1 out of 8 investigated subjects. This indicates that such high loads are common in reality. Therefore presentation and discussion of the loads is focused on the HIGH100 loads. The AVER75 pattern can be obtained from the HIGH100 pattern by multiplication with the factor C_aver_. A low C_aver_ value indicates a high variation in F_res_ between the investigated subjects. A C_aver_ value of 50%, for example, would indicate that, for the same activity, the peak value of F_res_ in one of the investigated subjects was twice as high as the average of all investigated subjects.

### Peak loads ‘PEAK100’ for subjects with high body weight

In the AVER75 patterns of F_res_, obtained from all the investigated subjects and all the loading cycles, that single trial was identified (T3 in Figure 1A) which had the absolute highest peak value P3. The load components from this trial were multiplied by 1.33 * F_P_ (Figure 1B). F_P_ was the quotient between the highest peak value of any trial (P3 in Figure 1A) and the inter-individual average of all subjects (P1 in Figure 1A). The obtained pattern was named ‘PEAK100’ and represents the absolute highest force F_res_ that could act during occasional trials in subjects with a BW of 100 kg. A high factor C_peak_ between the HIGH100 and the PEAK100 loads indicates that the variation of the HIGH100 loads from trial to trial is large.

### Extreme load components ‘EXTREME100’ for subjects with high body weight

The procedures described above, used to define the standardized average, high and peak loads, solely depend on the analysis of the resultant force F_res_ and its peak values. Therefore, all load components in the AVER75/PEAK100 data only differ by the factors C_aver_/C_peak_ from the same components in the HIGH100 data. This means that the load directions during the whole loading cycle are the same for each of the 3 load levels. When testing wear or strength of implants, the load directions in addition to the load magnitudes influence the results. A smaller force can be more detrimental than a higher force when it acts in a different direction, for example.

The peak values of some components vary intra-individually much more than F_res_. This indicates that the resultant force and/or moment acts in directions which can deviate greatly from the directions determined by the average components. Such effects cannot be detected when only analyzing the average force and moment components. Therefore, selected relative minima maxima in the time courses of the 6 load components were specified and their lowest/highest values were determined from the data of all subjects and all single trials. Included in this analysis were the data generated from both measurement sessions (Table 2), to increase the number of evaluated trials. The obtained values were named the ‘EXTREME100’ load components. Extreme values of single components may be suited for analyzing the mechanical reasons of *untypical* implant failures due to loosening, excessive wear, breakage or other factors.

### Knee flexion angle

The 3D kinematics of each subject's lower limbs were measured using reflective markers attached to the skin and tracked at 120 Hz using a 12-camera motion capture system (Vicon, Oxford, UK). The marker set consisted of 46 markers placed on the subjects' legs and pelvis [26]. The method used for determining the skeletal kinematics has been described in detail previously [27].

The same warping paths, obtained when averaging the resultant force F_res_ from single cycles or subjects, were applied to the synchronously measured knee flexion angle. The obtained flexion-time patterns are valid for all standardized loads (AVER75, HIGH100, and PEAK100).

## Results

**Figure 2:**
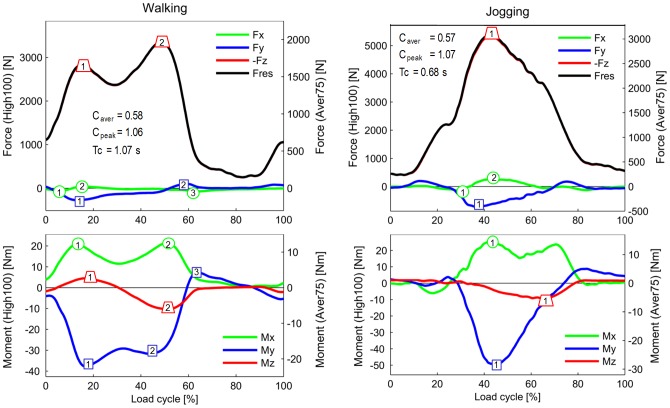
Forces and moments during walking and jogging. Left scales = high loads HIGH100. Right scales = average loads AVER75. Top diagrams = force components and resultant force. Bottom diagrams = moment components. Symbols with numbers = peak values for which the ranges of the ‘EXTREME100’ are listed in Table 3. C_aver_ = factor used to convert all HIGH100 load components to AVER75 components. C_peak_ = factor used to convert all HIGH100 load components to PEAK100 components. Tc = average cycle time. Data averaged for 8 subjects and all trials. Jogging data from only 3 subjects. Because –F_z_ is nearly identical to F_res_, the curve of –F_z_ is mostly invisible.

**Figure 3:**
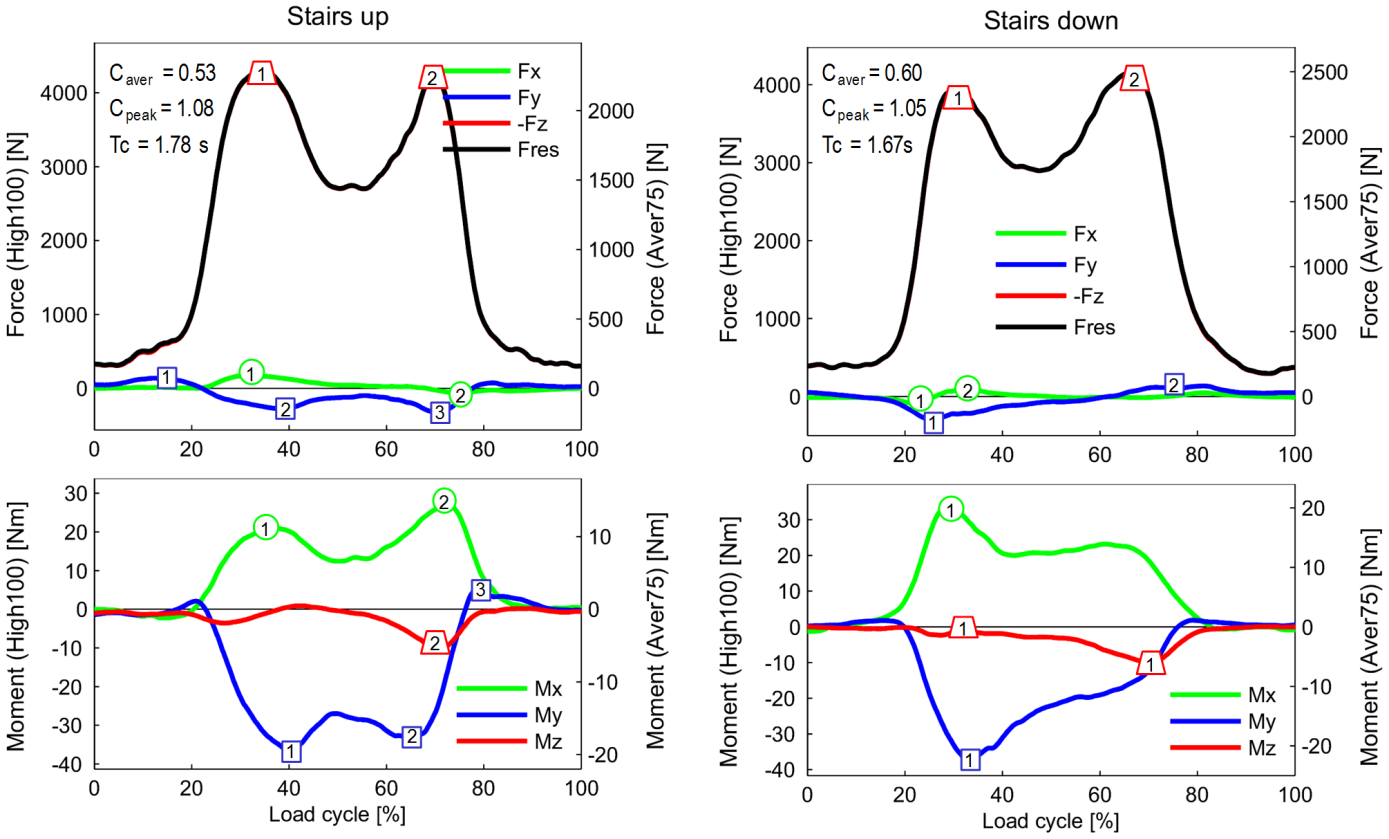
Forces and moments during ascending and descending stairs. For explanations, see Figure 2.

**Figure 4:**
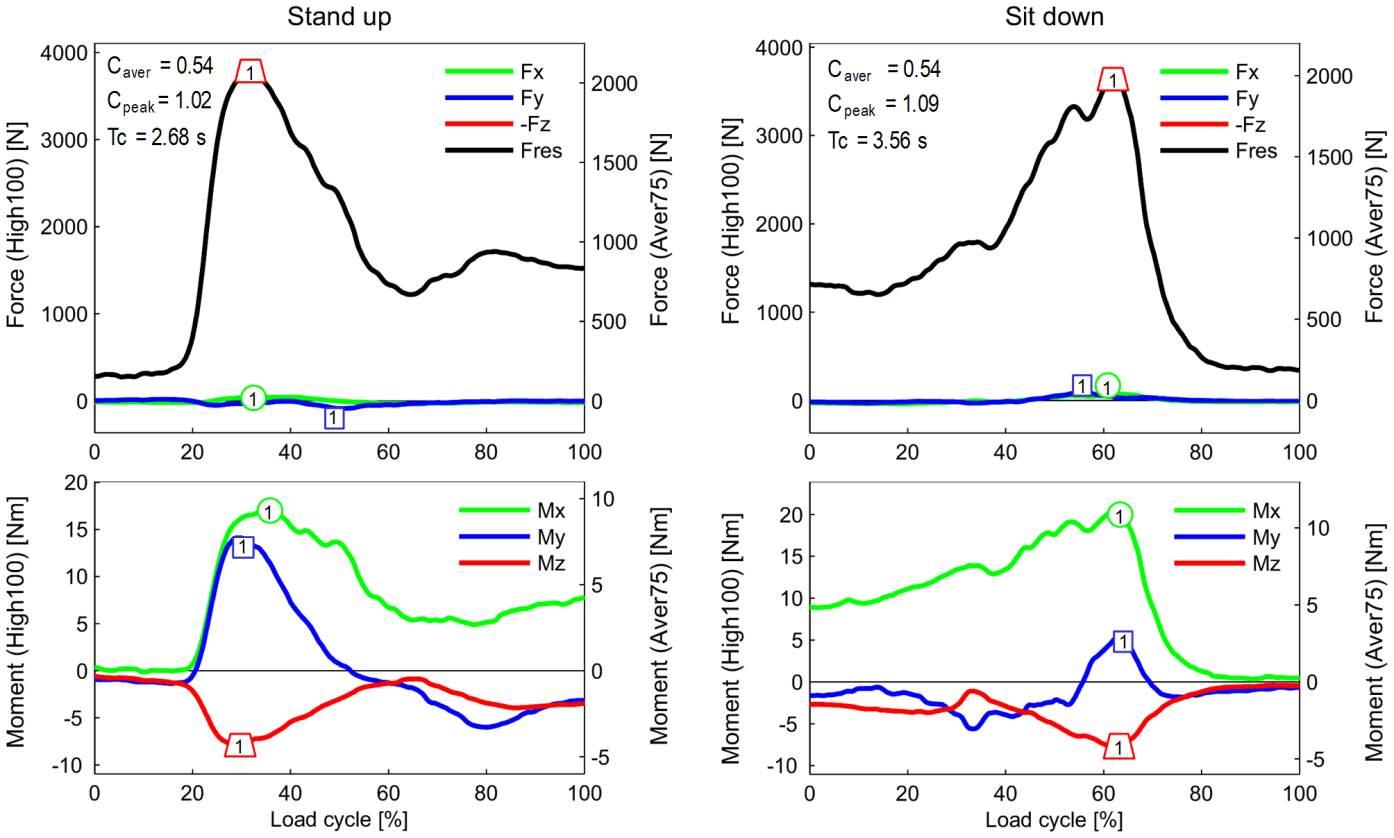
Forces and moments during standing up and sitting down. For explanations, see Figure 2.

**Figure 5:**
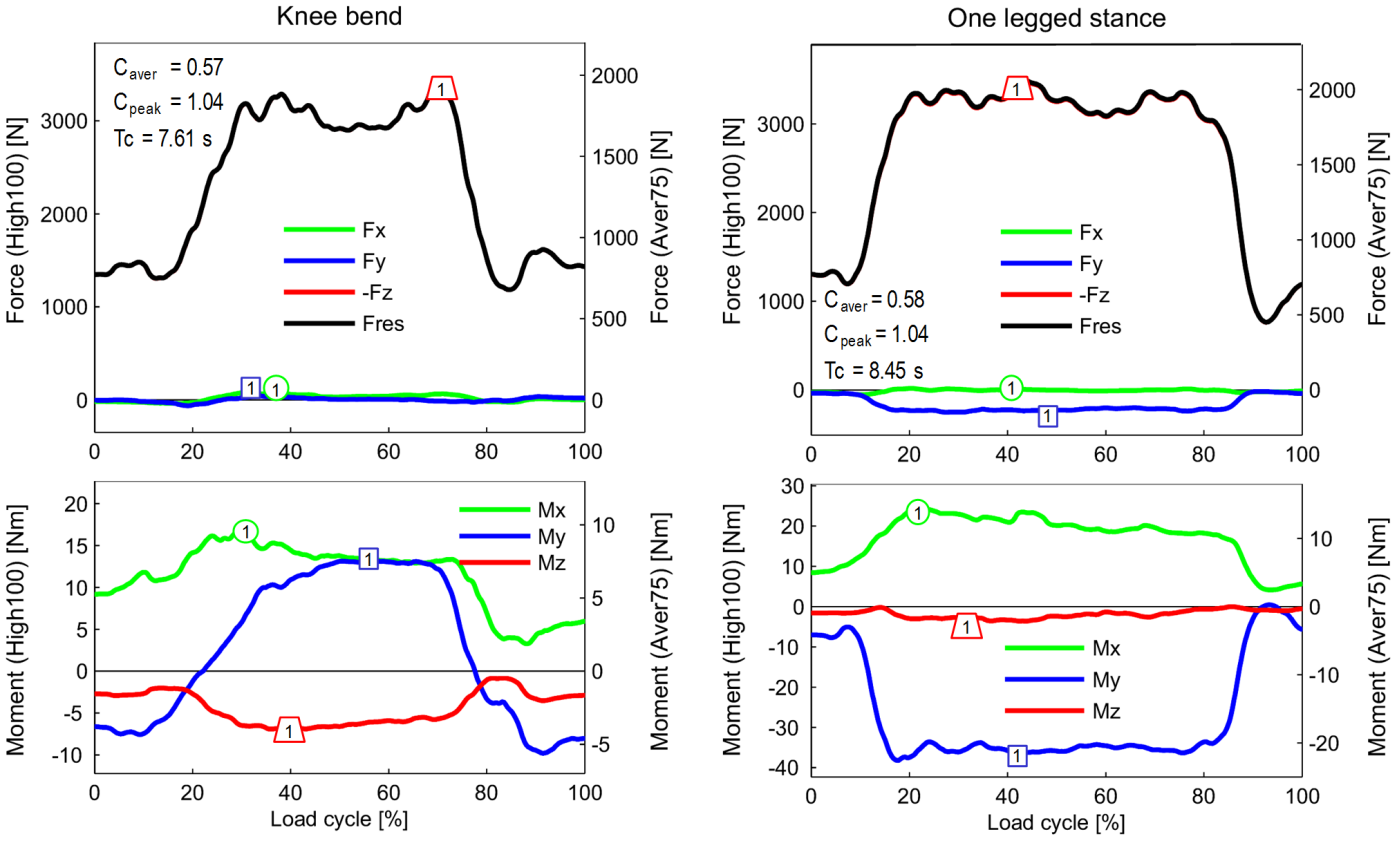
Forces and moments during knee bend and one-legged stance. Diagrams start and end with two-legged stance. For more explanations, see Figure 2.

All values of the load components and their resultants, stated in the following sections, refer to the HIGH100 loads. The HIGH100 data, collected during the different activities, are charted in the diagrams of Figures 2 to 5 with the left scales. Additional right scales allow reading the AVER75 data from the same diagrams. The C_aver_ and C_peak_ values, required for calculation of the AVER75 and PEAK100 loads from the HIGH100 loads, are listed in Table 2 and indicated in Figures 2 to 5. Table 2 also lists the average cycle times T_c_ from the data collected at the second postoperative date.

### Resultant force F_res_ and axial force component –F_z_ (upper diagrams in Figures 2 to 5)

**Figure 6:**
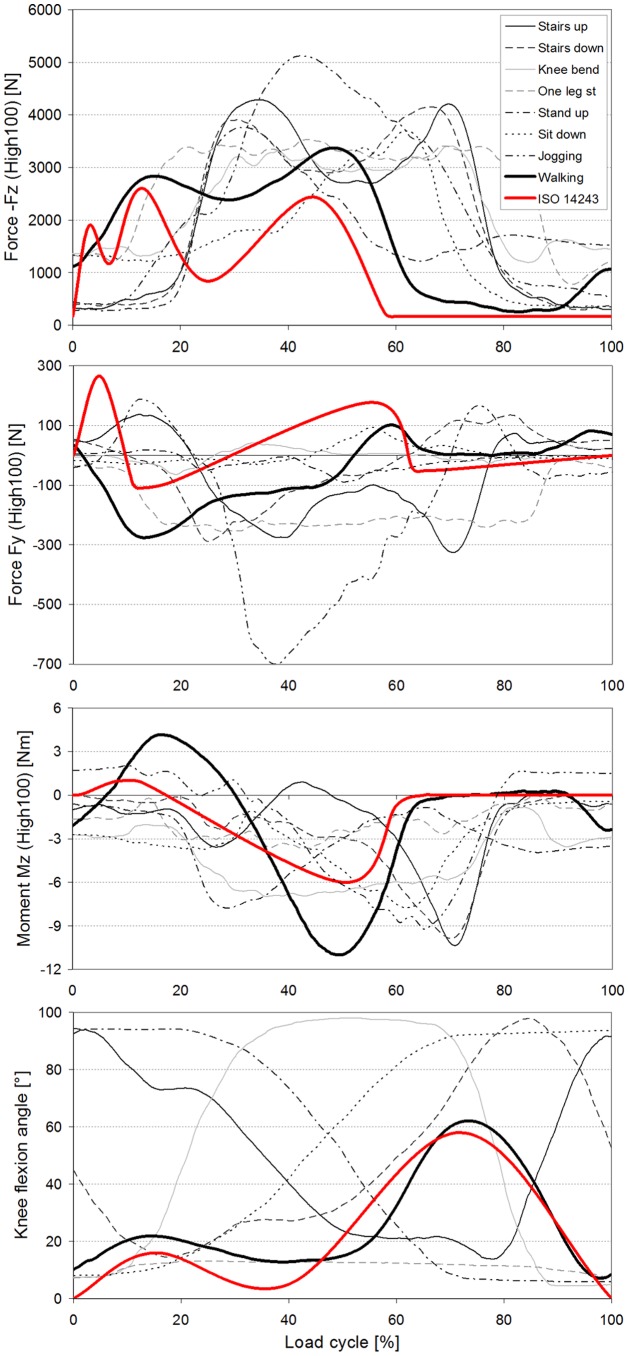
Comparison of measured load components and knee flexion angle with wear test standard. Average time courses of measured HIGH100 load components –Fz, Fy, Mz, and knee flexion angle during all investigated activities and comparison with ISO 14243 wear test standard.

Because the negative axial component –F_z_ always nearly equals F_res_, the data and findings for *F_res_* can approximately be transferred to –F_z_. When comparing the highest forces from all investigated activities except jogging, it becomes obvious that their peak values are very close together, encompassing a range of 3,372–4,218N (Figure 6).

During walking and ascending or descending stairs, F_res_ always had two maxima during each loading cycle. During walking, the second peak, which occurred at the instant of contralateral heel strike (3,372N), was larger than the first peak, at the instant of contralateral toe off (2,848N). During ascending or descending stairs, both peaks were higher than the peaks that occurred during walking. Their magnitudes had all similar values between 3,718 and 4,218N. During the one-legged stance, F_res_ reached a height similar to that of the second peak during walking.

The peaks of F_res_ during exercises with 2-leg support did not deviate much from the peaks that occurred when only one leg temporarily supported the whole BW. Rising from a chair with a maximum knee flexion angle (KF) of 94° or sitting down (94° KF) caused nearly the same peak values (3,792 and 3,697N, respectively). During the knee bend exercise, the peak was lower (3,407N) than the peak that occurred during the rising from a chair exercise, although the knee was flexed slightly more (98° KF).

During jogging, only one force maximum was observed. The peak force of 5,165N was 53% higher than the maximum force which acted during walking.

When the AVER75 forces F_res_ were expressed in %BW, we obtained 226/267%BW for the 1./2. peak during walking, 311/305%BW (1./2. peak) when ascending stairs, and 280%BW (maximum) when rising from a chair. The forces F_z_ had nearly the same values.

### Transverse forces F_x_ and F_y_ (upper diagrams in Figures 2 to 5)

**The** medial-lateral force F_x_ was small during all investigated activities. Except for jogging, the forces in the medial direction (F_x_<0) were always smaller than 100N. Force values higher than 100N in the lateral direction (F_x_>0) were only observed when ascending stairs (167N) or jogging (246N).

The peak values of the anterior-posterior force F_y_ were always larger than those of F_x_. During walking, ascending and descending stairs, as well as during the one-legged stance, peak values of F_y_ nearly always acted in the posterior direction (F_y_<0). With a range of −255N to −326N, the peak values had similar magnitudes for all 4 activities. The highest force recorded in the posterior direction was −699N and occurred during jogging.

The forces recorded in the anterior direction (F_y_>0) were generally much smaller than those acting in the posterior direction. Forces between 102N and 137N were recorded during walking and during ascending or descending stairs. The highest values, up to 189N, were measured during jogging. Although the flexion angles during knee bends and when sitting down or standing up were higher than during the other activities (Figure 6), the positive forces F_y_ stayed very low and did not exceed 94N. Alternating directions of F_y_ within the same loading cycle and values above 100N were only found during walking, climbing stairs, and jogging.

When expressed in %BW instead of N, the peak shear forces F_z_ of the AVER75 data were −32/+15%BW (1./2. peak) during walking, +14/−54%BW (1./2. peak) during climbing stairs and −19/+10%BW (minimum/maximum) during the chair rise exercise.

### Torsional moment M_z_ (lower diagrams in Figures 2 to 5)

High M_z_ values, due to an outwards rotation of the tibia (M_z_>0), were only found during walking at the instant of contralateral toe off. Throughout the entire loading cycle of all of the other activities, M_z_ was close to zero or negative, even during jogging. The tibia then rotates or tries to rotate inwards. During all activities except the one-legged stance, the peak values of M_z_ were between −7.0 and −10.5 Nm. The largest negative torque was measured during walking at the instant of contralateral heel strike, and it was even higher than the torque measured during jogging. Walking was the only activity during which a moment M_z_ of non-negligible magnitude acted in alternating directions.

### Transverse moments M_x_ and M_y_ (lower diagrams in Figures 2 to 5)

Although the knee movement changes between flexion and extension during all activities except standing, the moment M_x_ in the sagittal plane was always positive or close to zero. Small, negative values were recorded shortly before heel strike during jogging only. Positive values of M_x_ during extension phases cannot be caused by friction, but are the result of a posterior shift of -F_z_. This shift causes a moment that counteracts and exceeds the friction moment. The positive patterns of M_x_ in the extension phases therefore indicate that such a posterior shift of the axial force occurs during all activities. Except for descending stairs, the peak values of M_x_ lay between 17 and 27 Nm. If friction around the x-axis is neglected, this corresponds to backwards shifts of -F_z_ by about 5 to 10 mm. If friction is realistically taken into account, the shift would be even larger. While descending stairs, the highest peak values (34 Nm) were measured.

While ascending or descending stairs and during the one-legged stance, the abduction moment M_y_ was negative throughout the whole loading cycle or at least most parts of it. This negative moment indicates an adduction of the tibia *or* a medial shift of -F_z_. The magnitudes of M_y_ were close to -40 Nm, corresponding to a shift of -F_z_ of approximately 10 mm if friction is neglected. Small, positive values of M_y_ were found during the extension phases of walking and jogging, but the highest magnitudes of M_y_ were then also negative, with values of −38 and −47 Nm, respectively. Alternating directions of M_y_ were measured during knee bends and when standing up or sitting down. When standing up, M_y_ was 2.7 times higher than when sitting down.

### AVER75 and PEAK100 loads (Table 2)

The multiplication factors C_aver_ or C_peak_ have to be applied to the HIGH100 loads to obtain the AVER75 or PEAK100 data. The AVER75 loads are much smaller than the HIGH100 loads. Depending on the activity, the AVER75 load values are only 53–60% of the HIGH100 loads. This indicates that the loads vary strongly inter-individually. The values of C_peak_ were between 1.02 and 1.09, i.e., the PEAK100 loads are no more than 9% higher than the HIGH100 loads.

### Inter-individual variations of load patterns (Figure 7)

Only examples of the variation of the load components between the investigated subjects can be given here. Data from all activities and subjects is accessible from www.OrthoLoad.com (menu Test Loads).

The time courses of F_z_ (and therefore also of F_res_) from the different subjects were relatively uniform for all activities, but there were large differences observed in the magnitudes. This difference in magnitudes can also be seen indirectly from the low values of C_aver_ (Table 2). For the cyclic activities of walking and jogging, the patterns of all of the components except F_x_ were relatively uniform. For all other activities, the time courses of F_x_, F_y_, and, to a lesser extent, the components M_x_ and M_y_ were extremely different between the subjects. The most pronounced inter-individual variations were found during the non-cyclic activities: standing, knee bends, and ascending and descending stairs.

### Extreme load components EXTREME100 (Table 3)

**Figure 7:**
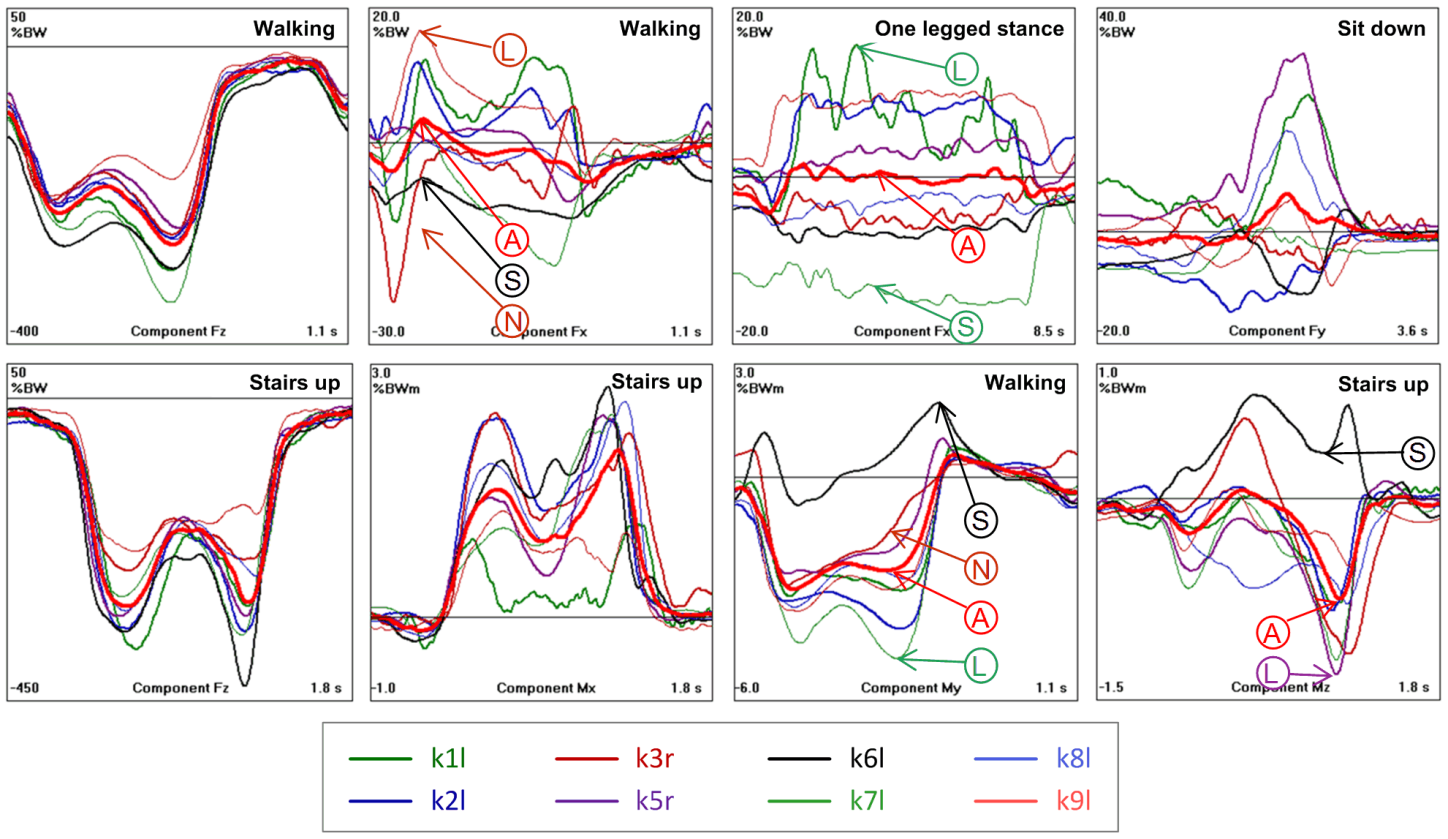
Load components with modest and strong inter-individual variations. Numbers are in %BW and %BWm (before normalization of the body weight to 75 kg to obtain the AVER75 loads). Thin lines = individual averages from 8 subjects. Thick lines = averages from all subjects. Top diagrams = force components. Bottom diagrams = moment components. Left 2 diagrams = similar patterns in all subjects. Right 6 diagrams: individually very different time courses. Even the signs of the highest extrema can differ. For further explanation, see the text.

Selected peak values of all load components were analyzed with respect to their extreme magnitudes, using data from all trials, all subjects, and from the two postoperative measurement sessions. The selected extrema are indicated and numbered in Figures 2 to 5. Because of the described inter-individual variations in the load patterns, the ranges of the selected peak values were sometimes difficult to determine (Figure 7). The average of a certain peak value (“A” in Figure 7) can be positive or negative. But in some subjects, the same peak value had an opposite sign (“S” in Figure 7), or did not even exist in others (“N” in Figure 7). These cases were excluded in the determination of the extreme peak values. The highest values of the relative maxima and the lowest values of the relative minima (“L” in Figure 7) are listed in Table 3.

**Table 3:** EXTREME100 forces [N] and moments [Nm].

Component	D	Walking	Ascending Stairs	Descending Stairs	OL stance	Stand. up	Sitting down	Knee bend	Jogging
**Fres**	**1**	3110	4209	4787	3676	3870	4036	3608	5551
	**2**	3581	4572	4348	-	-	-	-	-
**Fx**	**1**	−294	307	−416	222	257	301	318	−423
	**2**	292	−283	308	-	-	-	-	697
	**3**	−209	-	-	-	-	-	-	-
**Fy**	**1**	−605	220	−565	−557	−266	392	324	−1148
	**2**	221	−679	368	-	-	-	-	-
	**3**	-	−438	-	-	-	-	-	-
**-Fz**	**1**	3100	4169	4776	3667	3867	4033	3605	5396
	**2**	3571	4552	4347	-	-	-	-	-
**Mx**	**1**	25.9	30.5	59.1	38.7	21.4	28.6	46.1	39.8
	**2**	32.2	36.0	-	-	-	-	-	-
**My**	**1**	−50.2	−48.8	−68.8	−57.3	25.1	22.8	23.3	−57.1
	**2**	−53.2	−55.1	-	-	-	-	-	-
	**3**	27.3	37.2	-	-	-	-	-	-
**Mz**	**1**	12.0	10.5	−18.5	−13.3	−11.4	−10.8	−13.9	−13.7
	**2**	−18.9	−13.9	-	-	-	-	-	-

The numbers # = 1, 2, and 3 of the extrema are indicated in Figures 2–5.

Values were derived from measurement dates 1 and 2 (column D). OL stance = one-legged stance.

The inter-individual variations of single load components can be estimated by comparing their ranges with the peak values indicated on the component curves in Figures 2 to 5. Three examples are given here: A) peak “2” of F_x_ during walking (Figure 2) had an average value of 45N, but an EXTREME100 value of 292N was measured in subject K1L (Table 3); B) peak “3” of M_y_ during walking (Figure 2) had an average value of 7.3 Nm, but had an EXTREME100 value of 27.3 Nm in subject K8L ; C) peak ‘2’ of F_y_ during ascending stairs (Figure 3) had an average value of −276N, but had an EXTREME100 value of −679N in subject K1L.

Deviations of a factor of 5 or more were frequently observed between the average and individual peak values, especially in the transverse force and moment components.

### Comparison of ISO loads with standardized loads

In Figure 6, the 3 load components defined by the ISO standard 14243 for wear tests are compared with the same components in the measured HIGH100 data from all activities.

### Comparison of ISO loads with data from walking

The ISO loads were defined to simulate walking. However, nearly all extrema in the time courses of the ISO components were smaller than the HIGH100 values. The 1^st^ small maximum in the ISO course of the axial force –F_z_ was lacking in reality. The 2^nd^ ISO maximum was only 9% smaller than the measured maximum, but the 3^rd^ maximum was 39% smaller. For the anterior force F_y_, the first ISO peak was lacking again *in vivo*, the 2^nd^ ISO peak was 158% smaller, but the 3^rd^ ISO peak was 43% larger than measured *in vivo*. The largest differences between the ISO standard and the values measured in this study were determined for the torsional moment M_z_. The 1^st^ ISO peak value was 287% smaller and the 2^nd^ ISO peak was 82% smaller than *in vivo*.

### Comparison of ISO loads with data from other activities

A direct comparison between the mechanical effect of the ISO standard loads and the measured *in vivo* HIGH100 loads is not possible because the peaks of the ISO components act at flexion angles that are different than the flexion angles measured during the activities investigated in this study (Figure 6, bottom). The *in vivo* maxima of -F_z_ were determined to be much higher than the ISO maxima during all investigated activities. For the 1-legged stance, knee bend, standing up and sitting down activities, the measured -F_z_ maxima were 31–46% larger than the ISO maxima. During ascending or descending stairs, the measured peaks were 60–65% greater than the ISO peaks, and during jogging, the measured maxima were 97% greater than the ISO maxima.

The measured forces in the anterior direction (F_y_>0) were larger than the 2^nd^ ISO maximum only during jogging (+7%). Posterior forces (-F_y_<0) that were larger than in the ISO standard were found during ascending (+196%) or descending (+163%) stairs, the one-legged stance (+132%), and jogging (+535%).

Except for walking, only during jogging did the measured torsional moment M_z_ have a higher maximum (+100%) than the ISO standard. The absolute values of the minima of M_z_ were higher in the measured *in vivo* values compared with the ISO values during standing up (+30%), sitting down (+28%), knee bends (+17%) and jogging (+53%).

## Discussion

### Limitations of the study

Even though the joint loads were collected from the largest group of subjects with instrumented knee implants currently available, the data would be different if more subjects were included in the study. In particular, the HIGH100 and PEAK100 loads would certainly increase. Deviating load levels can also be expected to occur in younger or very old subjects. Although the literature shows that in 2002 only 2.3% of people had a BW higher than 100 kg, this percentage may grow in the future. If that is the case, the loads reported here may even be exceeded.

### Comparison with previous data

The only *in vivo* knee loads of other authors which can be compared with our data were measured with two different instrumented tibial trays [14], [15]. In studies with 1–3 subjects axial forces of 180–280%BW were measured during walking, 250–260%BW during chair rise, 250–300%BW when ascending and approximately 350%BW when descending stairs [16]–[18], [28], [29]. Peak anterior shear forces of 30%BW during walking, 26%BW during stair climbing, 17%BW during chair rise, and 15%BW during squatting were previously reported from one subject [18].

The peak AVER75 values which we determined for F_res_ and F_y_ are in the same range as the values determined in these previous studies. However, the large individual variation of F_z_ which we found (www.OrthoLoad.com, menu Test Loads) could not have been determined in these publications, so no further comparisons could be made.

Our actual data slightly deviate from previous own measurements in only 5 of the subjects, taken at an earlier postoperative time [1]. Previously the average resultant forces were by −3% (walking), +8% (going up stairs), −0.5% (going down stairs), −11% (standing up), −8% (sitting down), and −3% (knee bends) different from the current AVER75 results. To prove whether the total force had indeed increased with the postoperative time during most activities, an analysis of the same sub-group would be required.

### Adaptation of reported loads to test conditions

In joint simulators, cyclic loads which must start and end at the same values and should have the same slope are applied. Due to the time warping procedure, used to average single load cycles, these requirements are not perfectly met in this study. Therefore, curve fitting procedures must be applied to connect the last and first parts of the loading cycles reported here. Because their start and end values do not deviate much, 2 or 3% of the cycle durations may be appropriate for these transitions. The loads during standing up and sitting down may be combined to achieve cyclic loads.

### Which loads for which test or analysis?

Our study shows large differences between measured loads, which can act in patients with a high body weight, and those defined in the ISO standard. Differences between this standard and analytically determined loads during walking have also been reported by others [22], [30], [31].

Some structural failures of knee implants and delamination of polyethylene, which occur *in vivo*, cannot be replicated by simulator tests [32]. When the ISO loads were replaced by a profile containing only 10% walking cycles, but 80% of cycles of ascending and descending stairs, plus cycles from chair raising and deep squatting, wear in an unicompartmental implant rose four times [33]. When neglecting either F_y_ or M_z_ in ISO tests, the wear rate dropped by 90% [34]. This indicates that wear would greatly increase if these components were higher. Under loads acting during activities of daily living, conventional polyethylene inlays had 30% higher wear rates than under ISO loads. If loads under high flexion were applied, the wear rate grew by 168% [31]. Such observations indicate that tests and analyses of replaced and natural knees should not be performed under pure walking conditions as defined by the ISO 14243 standard. Instead, more realistic loads from walking should be chosen and other activities should be included, especially those requiring high flexion angles. A more strenuous loading profile has also been proposed by others [32], [35]. In light of these observations, the ISO wear test standard is presently discussed and will be modified in the future.

For testing or analyzing knee implants, the HIGH100 loads presented here (with fitted start and end intervals) should be chosen. For investigating problems of the static strength of the implant, its bony fixation, or of the surrounding soft tissues, the PEAK100 loads should be applied instead, but these are only 2–9% larger than the HIGH100 values. Small implants might not be able to withstand such high loads, and it could be discussed whether they are better tested at lower load levels.

### Replacement of single HIGH100 components by extreme EXTREME100 components

Except for the time courses of the HIGH100 loads, the most important finding of this study is the strong inter-individual load variation, especially of the transverse force components (Figure 7 and extended data from www.OrthoLoad.com). Due to the extreme variations of some load components, even the reported HIGH100 loads will most likely not suffice to explain every case of implant damage or failure of a surgical procedure. Overloading of polyethylene or of soft tissues, such as cruciate ligaments, may greatly depend on the magnitude of a single load component such as the a/p force F_y_. As shown here, these components can be much higher than in the time courses given by the HIGH100 data.

If a single component is suspected to cause a certain failure or contribute to it, it could be increased so that its peak value(s) corresponds to the EXTREME100 peak value (Table 3). It could be, however, that a failure is caused (or expected) by a combination of 2 or more extreme load components. The torque M_z_, for example, may be more detrimental if the axial force -F_z_ is small. In such cases, a large number of possible combinations with increased (or possibly decreased) components must be applied. This may be performed in analytical studies, but is difficult or even impossible in experimental investigations.

Another solution for this problem could be to increase all components during sections of the cycle time so that the marked extrema (Figures 2 to 5) reach the EXTREME100 values (Table 3). For peak “1” of F_res_ during walking (Figure 2, top left diagram), peak “2” of F_x_ would then have to be increased to 292N, peak “1” of F_y_ to −605N, and peak “1” of −F_z_ to 3,100N. This would, however, also change F_res_, which would increase from 2,848 to 3,172N. Furthermore, the loading directions would also be influenced (which may be the cause of the investigated implant damage). The frontal-plane angle between F_res_ and the z-axis, for example, would change from 0.9° to 5.4°. In the horizontal plane, the angle between F_res_ and the x-axis would decrease from 80.8 to 64.2°. The application of such a strategy is also questionable because the EXTREME100 values were taken from data collected from different subjects and may possibly never act combined in the same person.

We have no optimal suggestion for defining generally applicable combinations of load components for the most severe loading conditions. This problem must remain for future discussions, but it may well be that certain extreme loading conditions act in some subjects and that these cannot be appropriately tested in simulators.

### Loads acting on implants of different design and in the natural knee joint

The investigated implant has an ultra-congruent polyethylene inlay and requires sacrificing both cruciate ligaments. Most of the forces in the transverse directions and possibly also of the moments M_x_ and M_z_ are therefore taken up by the implant. If prostheses of different designs are implanted, for example with a moving platform, or models which retain the posterior cruciate ligament [5], [14], [15], [17], [18], unknown portions of these components will not be taken up by the implant but by the ligaments. Similar differences will occur between the loads acting in the instrumented implants and in natural joints.

The best method for determining how much of the loads are taken up by the soft tissues would be setting up a realistic finite element model of the natural or replaced knee, including the soft tissues and the patella and to apply the reported loads from the femur to the tibia.

### Medial-lateral force distribution

The distribution of the axial tibial Force –F_z_ between the medial and lateral compartment can easily be calculated [36] from the data which is accessible from www.OrthoLoad.com (Menu Test Loads). In a previous study [37] with 5 of the subjects investigated now, up to 85% of the peak force were transferred on the medial side, depending on the valgus angle of the knee. With regard to an even load distribution, a slight valgus angle of 2° to 3° would therefore be favorable.
